# Associations between Digital Cognitive Assessment and Amyloid Burden in Cognitively Unimpaired Older Adults

**DOI:** 10.1002/alz70857_106973

**Published:** 2025-12-26

**Authors:** Julia A. Sinople, Em Teixeira, Charles Denby, Nicholas Constant, Kunal Mankodiya, Geoffrey Tremont, Brian R. Ott, Lori A. Daiello, Jonathan D. Drake, Peter J. Quesenberry, Jill A. Kreiling, Laura E. Korthauer

**Affiliations:** ^1^ Rhode Island Hospital, Providence, RI, USA; ^2^ Well‐Aware, West Warwick, RI, USA; ^3^ University of Rhode Island, North Kingstown, RI, USA; ^4^ Warren Alpert Medical School of Brown University, Providence, RI, USA; ^5^ Brown University, Providence, RI, USA

## Abstract

**Background:**

Digital cognitive screening is poised to improve early detection of Alzheimer's disease and related dementias (ADRD), but existing tools are relatively insensitive to subtle cognitive changes in the preclinical stage of disease. Our digital assessment, the Rhode Island Mobile Cognitive Assessment Tool (RIMCAT), distinguishes cognitively impaired (MCI/early dementia) and unimpaired older adults with high sensitivity and specificity, but it has not yet been examined in the context of AD biomarker status. The goal of this study was to 1) enhance RIMCAT's sensitivity to early/preclinical AD risk by examining four cognitive paradigms sensitive to brain areas affected earliest in the course of disease; and 2) to examine associations between digital assessments and plasma amyloid Ab42/40 levels in cognitively unimpaired older adults.

**Method:**

Potential participants were cognitively unimpaired older adults currently enrolled in an ongoing longitudinal study of cognitive aging. We invited a random sample (*N* = 41) to participate, and 24 individuals (*M* age = 73 years, *M* education = 16 years, 54% male) were enrolled. Plasma samples were analyzed on the PrecivityAD platform to yield an amyloid probability score (APS; based on APOE status and Ab_42‐40_). Eleven (18% e4 carrier) had an APS < 35, suggesting low amyloid burden, and 13 (92% e4 carrier) had an APS >58, suggesting elevated amyloid burden. Participants completed RIMCAT and four new computerized subtests assessing perceptual discrimination, familiarity memory, feature binding, and response inhibition.

**Result:**

Participants in the elevated amyloid group performed worse on the RIMCAT and perceptual discrimination, familiarity memory, and feature binding tasks (non‐parametric Mann‐Whitney U tests *p*'s < .05; medium to large effect sizes, *r* = .28‐.51) compared to the non‐elevated amyloid group. When examined as a continuous variable across all participants, higher Ab42/40 ratio (less amyloid pathology) was associated with better performance on RIMCAT (*r* = .27) and all four new subtests (*r* = .23‐.40).

**Conclusion:**

We demonstrated the preliminary sensitivity of novel digital cognitive screening measures to amyloid burden in cognitively unimpaired older adults. Future research will validate these findings in a larger sample and examine associations with tau and other AD biomarkers.

